# Hydrophilic versus hydrophobic implant coatings: A comparative osseointegration study

**DOI:** 10.6026/973206300223289

**Published:** 2026-06-30

**Authors:** Ravi Ranjan Sinha, Sandeep Kashyap, Gourav Kumar Sahu, Gurdeep Kaur Chauhan, Pragati Mishra, Mayadevi Mayadevi

**Affiliations:** 1 Department of Prosthodontics Crown and Bridge, The Oxford Dental College, Rajiv Gandhi University of Health Sciences, Bengaluru, Karnataka, India; 2 Department of Dental Surgery, Sikkim Manipal Institute of Medical Sciences, Sikkim Manipal University, Gangtok, Sikkim, India; 3 Department of Conservative Dentistry and Endodontics, RKDF Dental College and Research Centre, RKDF University, Bhopal, Madhya Pradesh, India; 4 Department of Orthodontics, Mishra Dental Clinic and Orthodontic Care, Bhubaneswar, Odisha, India; 5 Department of Prosthodontics and Implantology, SB Patil Institute of Dental Science and Research, Rajiv Gandhi University of Health Sciences, Bidar, Karnataka, India

**Keywords:** Osseointegration, hydrophilic implant surface, hydrophobic implant surface, dental implants, implant stability

## Abstract

Achieving rapid and stable osseointegration remains a persistent challenge in dental implantology, particularly with variations in implant surface properties. Therefore, it is of interest to compare the effects of hydrophilic and hydrophobic implant surface coatings on osseointegration using clinical and radiographic parameters. Hence, a prospective comparative design involving 100 patients showed that hydrophilic implants yielded significantly higher implant stability (ISQ values) and reduced peri-implant bone loss over a 6-month follow-up period. Improved peri-implant soft tissue health, evidenced by reduced probing depth and bleeding on probing, was also observed in the hydrophilic group. Thus, we report hydrophilic surface modification as a superior strategy for enhancing early osseointegration and improving overall implant prognosis.

## Background:

Dental implants have become a widely accepted and predictable treatment modality for the replacement of missing teeth, owing to their high success rates and ability to restore both function and aesthetics [1]. The long-term success of dental implants largely depends on the process of osseointegration, which refers to the direct structural and functional connection between living bone and the surface of a load-bearing implant. Achieving rapid and stable osseointegration is crucial for reducing healing time, improving implant stability and ensuring long-term clinical outcomes. Over the years, significant research has focused on modifying implant surface characteristics to enhance this biological interaction [2]. Among the various surface properties, surface chemistry, topography and wettability play a vital role in influencing the biological response at the bone-implant interface [1]. Wettability, in particular, has gained considerable attention as it directly affects protein adsorption, cellular adhesion and subsequent bone formation. Implant surfaces can generally be classified as hydrophilic or hydrophobic based on their interaction with water. Hydrophilic surfaces exhibit a high affinity for water, promoting better spreading of biological fluids, while hydrophobic surfaces repel water and may limit initial biological interactions [4]. Hydrophilic implant surfaces are designed to enhance the early stages of osseointegration by improving the adsorption of proteins such as fibrinogen and fibronectin, which are essential for cell attachment [5]. These surfaces facilitate the migration, proliferation and differentiation of osteoblasts, thereby accelerating bone formation. Studies have demonstrated that hydrophilic surfaces can lead to faster healing times and improved primary stability, particularly in challenging clinical situations such as poor bone quality or immediate loading protocols. Additionally, increased surface energy in hydrophilic implants promotes a more favorable biological environment, leading to enhanced bone-to-implant contact (BIC) [6]. In contrast, hydrophobic implant surfaces, although traditionally used, tend to have lower surface energy and reduced wettability. This can result in less efficient protein adsorption and delayed cellular response. However, hydrophobic surfaces are not entirely disadvantageous; they may offer benefits in terms of reduced bacterial adhesion in certain conditions and improved shelf stability due to lower reactivity [7]. Nonetheless, their limitations in promoting rapid osseointegration have led to the development of advanced surface modification techniques aimed at improving their biological performance [8]. Various methods have been employed to modify implant surfaces to achieve desired wettability characteristics. Techniques such as sandblasting, acid etching, and plasma spraying, anodization and chemical modifications have been widely used to alter surface roughness and chemistry [9]. More recently, surface coatings with bioactive materials such as calcium phosphate, hydroxyapatite and titanium dioxide have been introduced to further enhance osseointegration. Additionally, ultraviolet (UV) treatment and storage in isotonic solutions have been utilized to increase surface hydrophilicity and maintain surface energy over time [10]. The interaction between implant surface properties and the biological environment is complex and influenced by multiple factors, including host bone quality, surgical technique and loading conditions. While hydrophilic surfaces have shown promising results in improving early osseointegration, the long-term clinical benefits compared to hydrophobic surfaces remain a subject of ongoing research [11]. Furthermore, variations in study design implant systems and evaluation methods have contributed to inconsistent findings in the literature [12]. Therefore, it is of interest to determine the comparative effectiveness of hydrophilic and hydrophobic implant surface coatings on osseointegration.

## Methodology:

This original research was designed as a prospective, comparative *in vivo* clinical study to evaluate the impact of hydrophilic and hydrophobic implant surface coatings on osseointegration. A total of 100 patients requiring dental implant therapy were selected based on prior literature and statistical power analysis to ensure adequate detection of intergroup differences. The participants were randomly allocated into two equal groups, with Group A (n = 50) receiving hydrophilic surface-coated implants and Group B (n = 50) receiving hydrophobic surface-coated implants and each patient received a single implant to eliminate clustering bias. The study population included individuals aged 20-60 years with partially edentulous sites requiring single-tooth implant placement, recruited from the outpatient department after obtaining informed consent. Only systemically healthy individuals with adequate bone volume, good oral hygiene (Plaque Index < 1) and non-smokers or light smokers (<10 cigarettes/day) were included, while patients with systemic conditions affecting bone healing, history of head and neck radiotherapy, pregnancy or lactation, parafunctional habits, poor oral hygiene, or active periodontal disease were excluded. Commercially available titanium implants with identical macro design and dimensions were used in both groups, differing only in surface characteristics, with hydrophilic-treated surfaces in Group A and conventional hydrophobic surfaces in Group B. All implant placements were performed under aseptic conditions following a standardized surgical protocol, involving administration of local anesthesia, crestal incision and osteotomy preparation as per manufacturer’s guidelines and implant insertion with controlled torque ranging from 35-45 Ncm to achieve optimal primary stability, followed by suturing and postoperative care instructions. Osseointegration was evaluated using both clinical and radiographic parameters at baseline, 1 month, 3 months and 6 months. Primary and secondary implant stability were assessed using resonance frequency analysis and expressed as Implant Stability Quotient (ISQ) values, while peri-implant bone loss was measured using standardized intraoral periapical radiographs with a paralleling technique. Additional parameters included healing time, determined by readiness for prosthetic loading and clinical indicators such as probing depth, bleeding on probing and absence of implant mobility or infection. Patients were followed up at 1, 3 and 6 months post-operatively, with comprehensive clinical and radiographic evaluations conducted at each visit and any complications were documented and managed appropriately. The collected data were statistically analyzed using appropriate software, with mean and standard deviation calculated for quantitative variables. Intergroup comparisons were performed using the independent t-test, while intragroup comparisons over time were analyzed using repeated measures ANOVA, with a p-value of <0.05 considered statistically significant. The study protocol was approved by the Institutional Ethical Committee and all procedures were carried out in accordance with ethical standards, with informed consent obtained from all participants prior to their inclusion in the study.

## Results:

A total of 100 patients (n = 100) completed the study, with 50 implants placed in each group. Healing was uneventful in the majority of cases and no implant failures were recorded during the 6-month follow-up period. The comparative evaluation of hydrophilic and hydrophobic implant surfaces revealed statistically significant differences in favor of hydrophilic surfaces, particularly in early osseointegration parameters. No statistically significant difference was observed between the groups in terms of age and gender distribution (p > 0.05), indicating comparability of baseline characteristics (Table 1). While baseline ISQ values were comparable, Group A demonstrated significantly higher ISQ values at 1, 3 and 6 months compared to Group B (Table 2). Hydrophilic implants exhibited significantly lower peri-implant bone loss at all-time intervals compared to hydrophobic implants (Table 3). Group A showed significantly better peri-implant soft tissue health, with lower probing depth and reduced bleeding on probing (Table 4). STATA regression analysis demonstrated that hydrophilic surface coating had a significant positive effect on implant stability (β = +3.72, p < 0.001) and a significant negative association with bone loss and probing depth (Table 5). The results of this study indicate that hydrophilic implant surfaces significantly enhance early and late osseointegration compared to hydrophobic surfaces. Improved implant stability, reduced marginal bone loss and better peri-implant soft tissue outcomes were consistently observed in the hydrophilic group. These findings were further supported by STATA-based statistical analysis, confirming the superiority of hydrophilic surface coatings in promoting favorable clinical outcomes.

## Discussion:

The present study evaluated the effect of hydrophilic and hydrophobic implant surface coatings on osseointegration and demonstrated that hydrophilic surfaces significantly enhanced implant stability, reduced peri-implant bone loss and improved soft tissue outcomes over a 6-month period. These findings suggest that surface wettability plays a critical role in early and intermediate phases of osseointegration, primarily by influencing protein adsorption, osteoblastic activity and bone-to-implant contact. The improved Implant Stability Quotient (ISQ) values observed in the hydrophilic group in this study are consistent with the biological principle that increased surface energy enhances cellular adhesion and bone formation. Hydrophilic surfaces facilitate the adsorption of key proteins such as fibronectin and fibrinogen, which promote osteoblast attachment and differentiation, thereby accelerating the healing cascade. In contrast, hydrophobic surfaces exhibit relatively reduced interaction with biological fluids, potentially delaying early osseointegration. The findings of the present study are in agreement with Lang *et al.* (2011), [13] who reported that hydrophilic (SLActive) implant surfaces demonstrated significantly greater bone-to-implant contact during early healing phases (2-4 weeks) compared to hydrophobic (SLA) surfaces. However, the differences diminished over time, suggesting that hydrophilic surfaces primarily enhance early osseointegration rather than long-term outcomes. Similarly, our study observed significant differences in implant stability and bone loss at early and intermediate time points, reinforcing the advantage of hydrophilic surfaces during initial healing. In another study, Siqueira *et al.* (2021) [14] investigated the role of hydrophilic titanium surfaces in compromised bone conditions such as osteoporosis. Their results indicated that hydrophilic surfaces modulated early osseointegration by enhancing bone formation and remodeling, even under unfavorable systemic conditions. This aligns with the present study, where hydrophilic implants showed superior outcomes despite normal physiological conditions, suggesting their potential advantage in both healthy and compromised bone. The experimental findings of Jinno *et al.* (2021) [15] further support the results of the current study. Their animal study demonstrated improved biomechanical stability and higher insertion torque values in hydrophilic implants compared to hydrophobic controls, indicating stronger bone anchorage. Correspondingly, our study reported significantly higher ISQ values in the hydrophilic group, highlighting enhanced mechanical stability and osseointegration. Additionally, Sánchez-Puetate *et al.* (2024) [16] evaluated bone formation around hydrophilic and hydrophobic implants in a murine model and found that hydrophilic surfaces resulted in improved bone volume, bone-to-implant contact and removal torque values, even under bisphosphonate therapy . These findings corroborate the reduced bone loss and improved peri-implant parameters observed in our study, emphasizing the beneficial role of hydrophilic surfaces in enhancing bone healing and integration. Furthermore, Pinto *et al.* (2023) [17] (evaluation in low-density bone models) demonstrated that hydrophilic implants exhibited significantly higher bone-to-implant contact and bone tissue formation compared to hydrophobic surfaces, particularly in low-density bone conditions. This is consistent with the present study, where reduced peri-implant bone loss was observed in the hydrophilic group, suggesting improved bone preservation and remodeling. Despite the consistent advantages observed with hydrophilic surfaces, it is important to note that some studies have reported that long-term differences between hydrophilic and hydrophobic surfaces may become less pronounced over time. This suggests that while hydrophilic surfaces accelerate early osseointegration, both surface types may eventually achieve comparable integration under favorable conditions. However, early stability is clinically significant, especially in cases involving immediate or early loading protocols. The present study adds to the existing body of evidence by providing clinical data with a standardized sample size and follow-up period. Unlike many previous studies conducted in animal models or controlled experimental settings, this study reflects real clinical conditions, thereby enhancing its applicability. The inclusion of multiple clinical and radiographic parameters, along with statistical validation, strengthens the reliability of the findings. However, variations in implant design, surface modification techniques and patient-related factors across different studies may account for minor discrepancies in outcomes. Additionally, the relatively short follow-up period in most studies, including the present one, limits the ability to draw conclusions regarding long-term implant success.

## Conclusion:

Hydrophilic implant surfaces showed significantly better osseointegration compared to hydrophobic surfaces. They showed higher implant stability and reduced peri-implant bone loss over time. Improved soft tissue response was also observed with hydrophilic coatings. Thus, we show the importance of surface wettability in early implant success. Hydrophilic surfaces may be preferred for achieving faster and more predictable clinical outcomes.

## Figures and Tables

**Table 1:** Demographic distribution of study population

**Parameter**	**Group A (Hydrophilic) (n=50)**	**Group B (Hydrophobic) (n=50)**	**p-value**
Mean Age (years)	38.6±9.2	40.1±8.7	0.412
Male (%)	28 (56%)	30 (60%)	0.689
Female (%)	22 (44%)	20 (40%)	

**Table 2:** Comparison of implant stability quotient (ISQ) Values

**Time Interval**	**Group A (Hydrophilic)**	**Group B (Hydrophobic)**	**p-value**
Baseline	68.4±3.5	67.9±3.8	0.521
1 Month	72.6±3.1	69.8±3.6	0.002*
3 Months	76.9±2.8	73.2±3.3	<0.001*
6 Months	79.3±2.4	75.6±2.9	<.001*

**Table 3:** Mean peri-implant bone loss (mm)

**Time Interval**	**Group A (Hydrophobic)**	**Group B (Hydrophobic)**	**p-value**
1 Month	0.42±0.10	0.55±0.12	0.001*
3 Months	0.68±0.14	0.89±0.16	<0.001*
6 Months	0.91±0.18	1.23±0.20	<0.001*

**Table 4:** Clinical parameters at 6 Months

**Parameter**	**Group A (Hydrophilic)**	**Group B (Hydrophilic)**	**p-value**
Probing Depth (mm)	2.1±0.4	2.6±0.5	0.003*
Bleeding on Probing (%)	6 (12%)	14 (28%)	0.041*
Implant Mobility	0	0	-

**Table 5:** STATA-Based statistical analysis summary

**Outcome Variable**	**Coefficient (β)**	**Std. Error**	**t-value**	**p-value**	**95% CI**
ISQ (6 months)	3.72	0.68	5.47	<0.001*	2.38 to 5.06
Bone Loss (6 months)	-0.32	0.07	-4.57	<0.001*	-0.46 to -0.18
Probing Depth	-0.51	0.16	-3.18	0.002*	-0.83 to -0.19
